# Antibacterial Action Mechanisms of Honey: Physiological Effects of Avocado, Chestnut, and Polyfloral Honey upon *Staphylococcus aureus* and *Escherichia coli*

**DOI:** 10.3390/molecules25051252

**Published:** 2020-03-10

**Authors:** Patricia Combarros-Fuertes, Leticia M. Estevinho, Rita Teixeira-Santos, Acácio G. Rodrigues, Cidália Pina-Vaz, Jose M. Fresno, M. Eugenia Tornadijo

**Affiliations:** 1 Department of Food Hygiene and Technology, Faculty of Veterinary Science, University of León, Campus de Vegazana, 24071 León, Spain; jmfreb@unileon.es (J.M.F.); metorr@unileon.es (M.E.T.); 2 CIMO, Mountain Research Center, Polytechnic Institute of Bragança, Campus Santa Apolónia, 5301-855 Bragança, Portugal; leticia@ipb.pt; 3 Division of Microbiology, Department of Pathology, Faculty of Medicine, University of Porto, 4099-002 Porto, Portugal; ritadtsantos@med.up.pt (R.T.-S.); agr@med.up.pt (A.G.R.); cpinavaz@med.up.pt (C.P.-V.); 4 CINTESIS—Center for Research in Health Technologies and Information Systems, Faculty of Medicine, University of Porto, 4200-450 Porto, Portugal; 5 Burn Unit, Department of Plastic and Reconstructive Surgery, Hospital São João, 4200-319 Porto, Portugal

**Keywords:** antibacterial mechanisms, flow cytometry, avocado honey, chestnut honey, polyfloral honey, *Staphylococcus aureus*, *Escherichia coli*

## Abstract

Numerous studies have explored the antibacterial properties of different types of honey from all around the world. However, the data available describing how honey acts against bacteria are few. The aim of this study was to apply a flow cytometry (FC) protocol to examine and characterize the primary effects of three varieties of honey (avocado, chestnut and polyfloral) upon physiological status of *Staphylococcus aureus* and *Escherichia coli* cells to reveal their antibacterial action mechanisms. The effects of honey samples on membrane potential, membrane integrity, and metabolic activity were assessed using different fluorochromes, in a 180 min time course assay. Time-kill experiments were also carried out under similar conditions. Exposure of *S. aureus* and *E. coli* to the distinct honey samples resulted in physiological changes related to membrane polarization and membrane integrity. Moreover, honey induced a remarkable metabolic disruption as primary physiological effect upon *S. aureus*. The different honey samples induced quite similar effects on both bacteria. However, the depth of bacteria response throughout the treatment varied depending on the concentration tested and among honey varieties, probably due to compositional differences in the honey.

## 1. Introduction

Global antibiotic crisis entails a threat not only for public health but also for food industry worldwide [1]. According to the last surveillance of antimicrobial resistance in Europe, *Staphylococcus aureus* and *Escherichia coli* currently present resistance to multiple antimicrobial groups [2], a disturbing fact since both bacteria are usually implicated in infections of community and healthcare settings and are amongst the foremost causative agents of food-borne infections [3].

The increasing emergence of multidrug-resistant pathogens stresses the need of developing alternative or complementary antimicrobial strategies. The demonstrated antibacterial activity of honey is attributed to various factors. Besides inherent physicochemical characteristics, such as high osmolarity or acidity, honey contains a wide variety of compounds with antimicrobial properties, among which hydrogen peroxide, polyphenols or methylglyoxal [4,5,6]. The presence and the quantity of antimicrobial factors in honey differ widely according to the floral source, harvesting season and geographical location, which may influence its overall effectiveness [4,7].

Numerous scientific studies have explored the antibacterial activity of different types of honey by itself or in combination with other antimicrobial agents as co-adjuvant [8,9,10,11,12]. However, very little is known about how honey acts against bacteria, and most of the studies focused on determining the action mechanisms of honey are referred to manuka honey (MkH) (*Leptospermum scoparium*) [13,14,15,16]. 

This study aimed to investigate the mechanism of antibacterial action of three varieties of honey (avocado, chestnut and polyfloral), evaluating the physiological status induced upon *S. aureus* and *E. coli* in a time course assay, assessed by flow cytometry (FC). In previous studies, these three honey varieties demonstrated higher antibacterial activity, as well as higher bioactive compounds concentration [17].

## 2. Results

### 2.1. Effects of Honey Samples on Cell Viability

**Figure 1:**
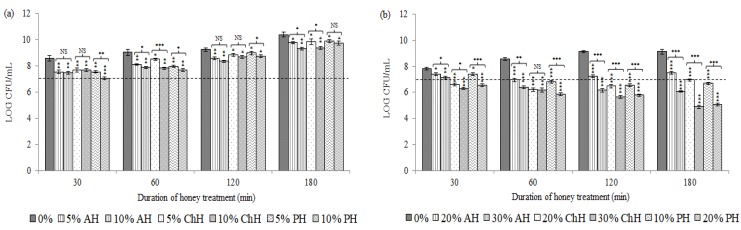
Effects of avocado honey (AH), chestnut honey (ChH) and polyfloral honey (PH) on *S. aureus* CECT 86 (**a**) and *E. coli* CECT 515 (**b**) cell viability assessed by colony forming units (CFU). Data at each time point corresponds to mean values of CFU counts ± standard deviation. NS indicate no significant differences and asterisks indicate significant differences (*: *p* < 0.05; **: *p* < 0.01; ***: *p* < 0.001) between treated cells *vs*. the control (non-treated cells) and between the two honey concentrations tested for each honey variety.

Figure 1 illustrates bacterial cell viability (*S. aureus* CECT 86 (a) and *E. coli* CECT 515 (b)) following the exposure to the two concentrations tested for each honey variety in comparison to non-treated cells control. 

Overall, significant differences (*p* < 0.05) in colony forming units (CFU) counts between honey-treated and non-treated bacteria in both species were observed. Similarly, different effects on bacterial cell viability were detected between the both honey concentrations tested, and between the honey varieties used, with some exceptions (Figure 1).

All types of honey at the both concentrations tested exhibited bacteriostatic effects upon *S. aureus* in the first 30 min of incubation (*p* < 0.01). After this time, cells recovered the ability to replicate and could grow progressively along the time up to the end of the treatment (Figure 1a). Nevertheless, CFU counts of honey-treated bacteria showed significant differences compared to non-treated bacteria cells (*p* < 0.05).

On the other hand, *E. coli* viability decreased progressively along the time, up to the end of the treatment at the higher concentrations tested (*p* < 0.01), whereas the lower concentrations displayed a general bacteriostatic effect along the treatment period (*p* < 0.05) (Figure 1b). These results demonstrated that honey concentration was a key factor upon *E. coli*.

### 2.2. Effects of Honey Samples on Cytoplasmic Membrane Potential

**Figure 2:**
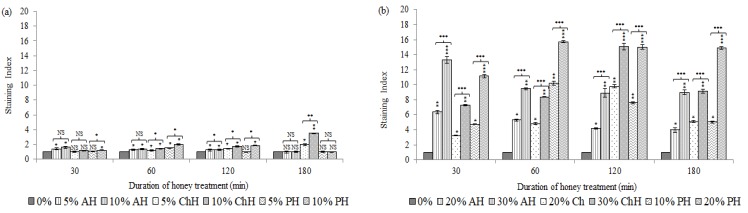
Effects of avocado honey (AH), chestnut honey (ChH) and polyfloral honey (PH) over membrane potential of *S. aureus* CECT 86 (a) and *E. coli* CECT 515 (b), evaluated using DiBAC4 (3) staining. Data at each time point corresponds to mean values of staining index ± standard deviation. NS indicate no significant differences and asterisks indicate significant differences (*: *p* < 0.05; **: *p* < 0.01; ***: *p* < 0.001) between treated cells *vs.* the control (non-treated cells) and between the two honey concentrations tested for each honey variety.

Figure 2 depicts the effect of the different honey samples on the cytoplasmic membrane potential of *S. aureus* CECT 86 (a) and *E. coli* CECT 515 (b) in contrast to non-treated bacteria control.

In general, the honey samples except for AH induced membrane depolarization on *S. aureus* after 60 min of incubation. However, *S. aureus* cells were able to recover from honey induced alterations, which effect seemed to be reversible after 120 min of exposure, except for ChH (Figure 2a). A minor, yet significant increase of fluorescence was registered soon after 30 min of incubation with both concentrations of AH (Staining Index (SI) at 5% (*w*/*v*) = 1.39; *p* < 0.05 and SI at 10% (*w*/*v*) = 1.61; *p* < 0.05) and with the higher concentration of PH (SI at 10% (*w*/*v*) = 1.20; *p* < 0.05). After 60 min of exposure all honey samples, at the both concentrations tested, induced bacterial membrane depolarization (*p* < 0.05). However, throughout treatment, bacteria exposed to AH and PH were able to progressively repolarize and recovered, at the end of the treatment, a similar membrane potential to viable untreated cells (*p* > 0.05). Conversely, treatment of bacterial cells with ChH resulted in a time-dependent increase of depolarized cells from 60 min (SI at 5% (*w*/*v*) = 1.21; *p* < 0.05 and SI at 10% (*w*/*v*) = 1.44; *p* < 0.05) up to 180 min of treatment (SI at 5% (*w*/*v*) = 1.97; *p* < 0.05 and SI at 10% (*w*/*v*) = 3.50; *p* < 0.01). Significant differences were observed between both treatment conditions with some exceptions. These factors suggest the relevance of honey concentration, treatment time, as well as the variety of honey, upon *S. aureus* cell membrane depolarization. 

Regarding *E. coli*, membrane depolarization occurred as an early event with all honey samples; a significant increase of fluorescence was registered soon after 30 min of incubation at the both concentrations tested (*p* < 0.05). However, from this time point up to the end of the treatment, the behavior of the bacterial cells differed according to the variety and the concentration of honey (Figure 2b). Bacteria treated with AH undergo a membrane depolarization from the first 30 min (SI at 20% (*w*/*v*) = 6.38; *p* < 0.01 and SI at 30% (*w*/*v*) = 13.32; *p* < 0.001). However, bacteria seemed to develop compensatory responses, which permitted to recover progressively the membrane potential. Nevertheless, the SI values were significantly higher than those of the viable untreated cells after 180 min (SI at 20% (*w*/*v*) = 3.97; *p* < 0.05 and SI at 30% (*w*/*v*) = 8.98; *p* < 0.01). Treatment with ChH resulted in a time-dependent increase of depolarized cells from 30 min (SI at 20% (*w*/*v*) = 3.25; *p* < 0.05 and SI at 30% (*w*/*v*) = 7.29; *p* < 0.01) up to 120 min of treatment (SI at 20% (*w*/*v*) = 9.79; *p* < 0.01 and SI at 30% (*w*/*v*) = 15.09; *p* < 0.001, respectively). After this time, cells seemed to partially repolarize under both treatment conditions (SI = 5.10; *p* < 0.05 and SI = 9.15; *p* < 0.01, respectively). Finally, cells exposed to PH displayed an increasing depolarization from 30 min (SI at 10% (*w*/*v*) = 4.74; *p* < 0.05 and SI at 20% (*w*/*v*) = 11.16; *p* < 0.01) up to 60 min of exposure (SI at 10% (*w*/*v*) = 10.21; *p* < 0.01 and SI at 20% (*w*/*v*) = 15.72; *p* < 0.001). At this time point, bacteria treated with 10% (*w*/*v*) of honey appeared to gradually recover membrane potential up to the end of the treatment (SI = 5.02; *p* < 0.05), whereas bacteria treated with 20% (*w*/*v*) of honey, kept similar values of SI up to 180 min (SI = 14.93; *p* < 0.001). Significant differences were observed between the two treatments, at all-time points monitored. These results show the relevance of honey concentration, treatment duration, as well as the honey type on cell membrane depolarization.

### 2.3. Effects of Honey Samples on Membrane Integrity

**Figure 3:**
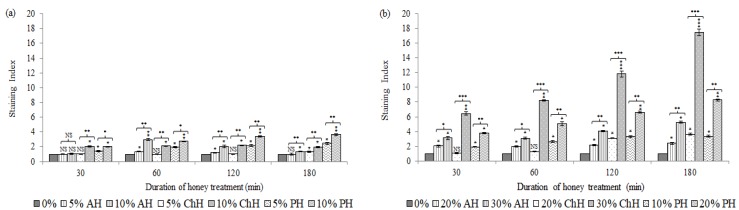
Effects of avocado honey (AH), chestnut honey (ChH) and polyfloral honey (PH) on membrane integrity of *S. aureus* CECT 86 (**a**) and *E. coli* CECT 515 (**b**), evaluated using propidium iodide staining. Data at each time point corresponds to mean values of staining index ± standard deviation. NS indicate no significant differences and asterisks indicate significant differences (*: *p* < 0.05; **: *p* < 0.01; ***: *p* < 0.001) between treated cells *vs*. the control (non-treated cells) and between the two honey concentrations tested for each honey variety.

Figure 3 showed the effect of honey upon bacterial membrane integrity of *S. aureus* CECT 86 (a) and *E. coli* CECT (b) compared with non-treated cells.

Overall, minor -yet significant- modifications in *S. aureus* membrane (Figure 3a) were observed. The effect exhibited a treatment time and honey concentration dependency. Cells treated with AH presented a significantly higher SI after 60 min of honey exposure with the both concentrations tested (SI at 5% (*w*/*v*) = 1.36 and SI at 10% (*w*/*v*) = 2.97; *p* < 0.05). However, along the treatment, bacteria seemed to recover progressively from this damage since the SI continuously decreased until the end of the treatment. At 180 min only the higher concentration of AH induced a significantly higher SI value comparing to untreated bacteria (SI = 1.35; *p* < 0.05). Bacteria treated with 5% (*w*/*v*) of ChH showed a significant PI-intake only after 180 min of treatment, whereas cells treated with 10% (*w*/*v*) displayed a significantly different SI value following 30 min of honey exposure, which was stable along the treatment. Finally, bacterial cells exposed to PH displayed, at both concentrations, a time-dependent increase of SI, from 30 min (SI at 5% (*w*/*v*) = 1.42; *p* < 0.05 and SI at 10% (*w*/*v*) = 2.01; *p* < 0.05) up to the end of the treatment (SI = 2.47; *p* < 0.05 and SI = 3.64; *p* < 0.01 respectively). Notably significant differences were also registered between both experimental conditions, what suggests that honey concentration, honey variety, as well as the treatment time are key factors influencing *S. aureus* membrane injury. 

Membrane alterations in *E. coli* were appreciated from the early stages of the treatment up to the end, in a clear time-dependent fashion (Figure 3b). Bacteria treated with 20% (*w*/*v*) of AH and 10% (*w*/*v*) of PH showed PI-uptake after 30 min (SI AH = 2.04; *p* < 0.05; SI PH = 1.92; *p* < 0.05), raising slightly up to the end of the treatment (SI AH = 2.40; *p* < 0.05; SI PH = 3.37; *p* < 0.05). Cells treated with 20% (*w*/*v*) of ChH showed SI values significantly different after 120 min of honey exposure (SI = 3.09; *p* < 0.05) increasing up to 180 min (SI = 3.64; *p* < 0.05). Bacterial cells treated with higher concentration of honey (30% (*w*/*v*) for AH and ChH, and 20% (*w*/*v*) for PH) resulted in a time-dependent increase of SI from 30 min (SI ChH = 6.44; *p* < 0.01; SI PH = 3.75; *p* < 0.05) or 60 min (SI AH = 3.08; *p* < 0.05) up to 180 min (SI AH = 5.26; *p* < 0.05; SI ChH = 17.45; *p* < 0.001; SI PH = 8.28; *p* < 0.01) of treatment. Significant differences were observed between both treatment conditions at all-time points, and for the three honey varieties. Honey concentration also played a key role, especially for ChH and PH.

### 2.4. Effects of Honey Samples on Metabolic Activity

**Figure 4:**
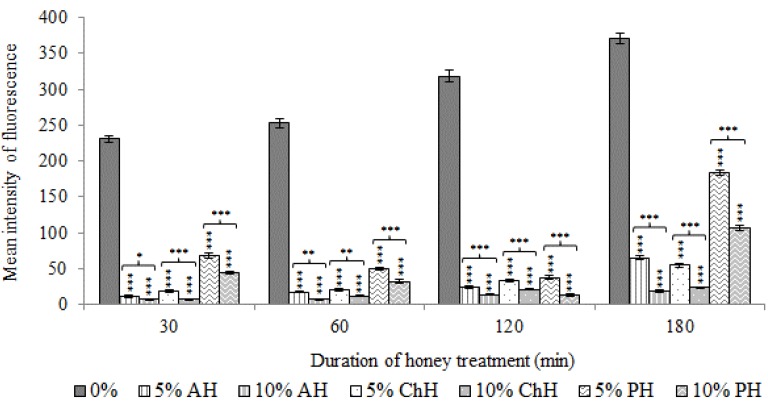
Effects of avocado honey (AH), chestnut honey (ChH) and polyfloral honey (PH) on *S. aureus* CECT 86 metabolic activity, evaluated using calcein-AM staining. Data at each time point corresponds to mean values of mean intensity of fluorescence (MIF)± standard deviation. NS indicate no significant differences and asterisks indicate significant differences (*: *p* < 0.05; **: *p* < 0.01; ***: *p* < 0.001) between treated cells vs. the control (non-treated cells) and between the two honey concentrations tested for each honey variety.

Figure 4 represents the metabolic effects of the different honey samples on *S. aureus* CECT 86 cells using calcein-AM. 

Mean intensity of fluorescence (MIF) values displayed by untreated cells increased slightly up for 180 min. However, bacterial cells exposed to honey showed a severe reduction on MIF values soon after 30 min of honey exposure (Figure 4). MIF values decreased, even with the lowest concentrations tested (MIF AH = 10.76 and 6.73; *p* < 0.001; MIF ChH = 17.99 and 6.69; *p* < 0.001 and MIF PH = 67.78 and 43.98; *p* < 0.001 at 5% and 10% (*w*/*v*), respectively). Nevertheless, different behavior was noticed among samples.

Fluorescence intensity increased progressively from 30 min until 180 min in bacteria ex posed to AH and ChH (MIF = 64.91 and 18.06; *p* < 0.001 and MIF = 54.63 and 23.72; *p* < 0.001, respectively at 5% and 10% (*w*/*v*) at 180 min). Conversely, PH induced a lower inhibitory effect on the metabolic activity of bacteria, although MIF values decreased progressively along to 120 min at the both concentrations tested (MIF = 37.48 and 12.75 respectively at 5% and 10% (*w*/*v*); *p* < 0.001). From this time point until 180 min of exposure, bacteria exhibited a recovery of metabolic activity at the both treatment conditions (MIF = 183.44 and 107.18; *p* < 0.001). Nevertheless, the MIF values of treated bacteria showed significant differences compared to non-treated bacteria cells (*p* < 0.001).

## 3. Discussion

Despite several reports on antibacterial properties of different varieties of honey [5,7,12,18,19], the majority of the studies investigating the action mechanism of antibacterial activity of honey have focused on MkH [20]. This study explored the effects of different varieties of honey upon *S. aureus* and *E. coli* physiology and aimed to determine the analogies or dissimilarities related to its antibacterial mechanisms, not only between them, but also with MkH, providing new insights regarding antibacterial effects of honey. The influence of the three honey samples tested (AH, ChH and PH) on bacterial cell viability, membrane potential, membrane integrity, and metabolic activity were assessed on *S. aureus* CECT 86 and *E. coli* CECT 515 reference strains. To accomplish this purpose, MLC and a higher concentration of each honey sample were tested.

The effect of honey on bacterial cell viability differed depending on the honey variety and the concentration tested. The lowest honey concentrations (20% (*w*/*v*) for AH and ChH and 10% (*w*/*v*) for PH) induced, in general, bacteriostatic effects on *E. coli* cells; whereas the highest concentrations (30% (*w*/*v*) for AH and ChH and 20% (*w*/*v*) for PH) produced the bacterial death This data suggests that honey concentration is an essential factor which determine the ability of *E. coli* cells to resist honey induced alterations. PH exhibited the most powerful repercussion on this bacterium.

On the other hand, no evidence of bactericidal activity upon *S. aureus* was found in the first 180 min of honey exposure despite using MLC and a higher concentration. The absence of bacterial growth found in the previous honey susceptibility tests [17] was understood as the bactericidal effect of honey, but indeed, it was not, as only bacteriostatic effects were observed in the first minutes of honey exposure. This fact could be explained, as some studies describe, by a phenomenon known as a viable but non-culturable (VBNC) state whereby bacteria lose the ability to grow on the routine media on which they normally grow [21,22]. A list of factors, both chemical and environmental, which have been reported to induce the VBNC state are varied and numerous [21,22] and suggest that this is an adaptive strategy for long-term survival of bacteria under unfavorable environmental conditions [23]. It is possible that honey samples, under the conditions in which the MIC and MLC values were determined (96-well microplates, without agitation and subsequent count of colony forming units on plates agar), could induce a VBNC state on *S. aureus*, elsewhere reported [21,24,25], and which was previously admitted as a lethal concentration. Although exposure to honey at 5% and 10% (*w*/*v*) did not reduce the growth rate of *S. aureus*, significant changes on physiological status of bacteria were observed; the same circumstance was previously described in *S. aureus* proteome when exposed to sub-inhibitory concentrations of honey [26].

Membrane potential plays an essential role in bacterial physiological processes. The loss of the equilibrium of ion concentration inside and outside of bacteria may affect their viability [27]. Honey-induced physiological changes on bacterial membrane potential were described for the first time in a previous study with MkH [28]. Similarly, in this study AH, ChH, and PH induced alterations upon *S. aureus* and *E. coli* membrane potential. However, membrane depolarization caused by these three honey types was greater and sooner than that caused by MkH at the same treatment conditions [28]. *S. aureus* cells exposed to 10% of MkH showed significant increase of fluorescence after 120 min of exposure [28], while at that concentration, AH and PH induced alterations after 30 min of exposure and after 60 min in the case of ChH. Similarly, *E. coli* cells exhibited a significant membrane depolarization after 120 min of treatment with 20% (*w*/*v*) of MkH and after 60 min at a higher concentration of MkH (30% (*w*/*v*)). Nevertheless, only 30 min were enough to induce a membrane depolarization on *E. coli* cells at similar concentrations of AH, ChH and PH. Under some treatment conditions, bacteria were able to gradually repolarize and recover, partially or completely, their membrane potential. Similar behavior was previously observed in *S. aureus* and *E. coli* exposed to MkH [28].

The loss of membrane integrity means a substantial damage for bacteria. The results obtained in this study reveal that treatment time and honey concentration are key factors regarding the induction of membrane injury in *S. aureus* and *E. coli*. Moreover, these results revealed that the distinct honey samples act differently between gram positive and gram negative bacteria, a fact which was already described in some previous studies with MkH [15,16,28,29]. While the effect of the different honey samples on *S. aureus* membrane integrity was limited, in the case of *E. coli,* the results obtained confirmed more meaningful effects. Under similar treatment conditions (honey concentration of 20% (*w*/*v*) tested with the four honey varieties), the three honey samples were able to cause a more relevant membrane integrity alterations than MkH [28]. However, this effect was more notable for PH, which evinced to be the most powerful sample.

The effects of the different honey samples on *S. aureus* metabolic activity were evaluated using calcein-AM. Only metabolically active cells are able to hydrolyze calcein-AM to a fluorescent compound by intracellular esterases. This compound accumulates inside cells with intact membranes and increases their fluorescence [30]. Considering the results obtained, AH, ChH, and PH induced a significant metabolism disruption on *S. aureus* cells as an early event. This effect could not be attributed to membrane pore formation, which may lead to the loss of fluorescence, since bacteria PI-intake was not significant until 60 min of exposure to AH and until 180 min of exposure to 5% (*w*/*v*) to ChH (Figure 3). In contrast, the less membrane integrity, the higher fluorescence intensity was observed with PH (Figure 3 and Figure 4). Slight differences observed between the two treatments were significant for all honey varieties, suggesting that metabolic inactivation is dependent from honey concentration, as well as from the treatment time. Our previous study confirmed similar consequences in *S. aureus* cells incubated with MkH [28]. However, metabolic disruption induced by MkH seemed to be an irreversible effect, whereas in this case, bacteria appear to reprogram their metabolism in response to the environmental stress, being able to progressively recover metabolic activity, at least partially, at the end of 180 min of honey exposure. AH and ChH exhibited a longer and more pronounced metabolic disruption. The transience of this effect might be overcome using higher concentrations of honey. Nevertheless, further studies are necessary to confirm this assumption. 

This study demonstrates that AH, ChH and PH were able to induce different bacterial compensatory responses. *E. coli* cells were not able to overcome from such an environmental stress, which resulted in the loss of bacteria viability when the highest concentration of each honey variety was used (20% or 30% (*w*/*v*) depending on honey type). On the other hand, *S. aureus* cells were capable of adapting to honey pressure, what might explain the reversibility of some of the effects and the increase in CFU counts throughout the treatment. However, considering the intricate relation observed between honey concentration and intensity of bacterial response, it is highly plausible that higher concentrations of honey could lead to a more pronounced effect and even bacterial death. To validate this hypothesis complementary further studies are necessary.

Inherent physicochemical properties of honey, as well as multiple compounds originated from the nectar of plants, pollen, propolis, and from honeybee itself, are responsible for its antibacterial activity [19]. Indeed, recent studies revealed that polyphenols are key components on antimicrobial effects of honey [7,11,19,31]. Phenolic compounds profiles are highly variable among different varieties of honey and specific floral markers of some monofloral varieties have been suggested [32,33,34]. These compositional variations could justify the differences in their bioactivity [17,35]. Previous studies confirmed that kynurenic acid and another unknown compound were present in higher concentrations in samples with a *Castanea sativa* origin [32,34]. Similar compounds were found not only in the chestnut, but also in the polyfloral honey samples tested in this study. Moreover, another two unknown compounds, probably kynurenic acid derivates, were found in both samples, in which *Castanea sativa* pollen was the predominant or secondary pollen-type found [17,36]. No specific phenolic compounds have been described in avocado honey. Nevertheless, the results obtained in this study and in a previous one, encourage the research of possible markers characteristic of this variety, which could explain its bioactive functions. In addition, the antimicrobial activity of many flavonoids has been attributed to different mechanisms, including cytoplasmic membrane damage, inhibition of cell wall, and cell membrane synthesis, inhibition of nucleic acid synthesis, and inhibition of energy metabolism [37,38]. Interestingly, some of these mechanisms are similar to those described in this study for honey as a whole, suggesting that flavonoids present in honey might be involved in these effects upon bacteria physiology. Nevertheless, honey compounds could function synergistically and in result of this complex blend, honey acts in a multifactorial way upon several cellular target sites [9,39,40]. Although in some cases AH, ChH, and PH presented, under the same treatment conditions, a greater effect upon bacteria than MkH, the truth is that the concentrations tested were not able to reduce bacterial viability in all cases. This fact suggests the existence of other antibacterial action mechanisms which could justify the irreversible effects of MkH.

## 4. Materials and Methods

### 4.1. Bacterial Strains, Growth Conditions and Inoculated Broth Preparation

*Staphylococcus aureus* (CECT 86) and *Escherichia coli* (CECT 515) from the Spanish Type Culture Collection were used as gram-positive and gram-negative bacteria reference strains, respectively. The kinetics of bacterial growth to define the bacterial exponential growth phase were previously determined [28].

A cell suspension in Mueller Hinton (MH; Sigma-Aldrich, St. Louis, MO, USA) broth with bacteria in initial exponential growth phase were prepared as was previously described by Combarros-Fuertes, Estevinho, Teixeira-Santos, et al. (2019) [28].

### 4.2. Honey Samples and Bacterial Susceptibility

Three samples of raw Spanish honey from different botanical and geographical origins [avocado honey (AH) and chestnut honey (ChH) from the Protected Denomination of Origin “Miel de Granada”, and an organic polyfloral honey (PH) from the province of León (Spain)] were used. These honey samples were previously characterized [36] and were selected from a total of sixteen samples because of their higher antibacterial effects [17].

MIC and MLC were previously defined as 5% (*w*/*v*) for *S. aureus* for all honey samples while for *E. coli* the MIC and MLC values varied depending on the honey variety: 20% (*w*/*v*) for avocado and chestnut honey samples and 10% (*w*/*v*) for polyfloral sample [17].

Stock solutions of 0.8 g of honey per mL in MH sterile broth were prepared to carry out the subsequent assays.

### 4.3. Evaluation of Cell Viability

The determination of bacterial cell viability was performed for both microorganisms by counting colony forming units (log CFU/mL) and following the protocol of a previous study with MkH [28].

The honey concentrations tested were in agreement with the susceptibility test results: 5% and 10% (*w*/*v*) for *S. aureus* and between 10% and 30% (*w*/*v*) for *E. coli* depending on the honey sample (10%–20% (*w*/*v*) for PH and 20%–30% (*w*/*v*) for AH and ChH). Bacterial suspensions (108 cells/mL) were exposed to honey samples for 30, 60, 120, and 180 min at 37 °C and 180 rpm. After honey treatment, bacterial suspensions were centrifuged, the supernatant was discarded, the pellets were washed, and the number of viable cells was determined following the protocol previously developed [28].

### 4.4. Functional Characterization of Honey-Induced Action

The physiological situation of bacterial cells after honey exposure in a time course assay was assessed by FC, following the previously optimized protocol with MkH [28]. Similar conditions to those used in the evaluation of cell viability were applied. After each treatment, bacterial suspensions were also centrifuged, the supernatant was discarded, and the pellets were washed with PBS to eliminate honey reminiscence. Afterwards, bacteria were homogenized and stained with PBS containing the different fluorochromes at the concentrations and the conditions detailed by Combarros-Fuertes, Estevinho, Teixeira-Santos, et al. (2019) [28].

The changes induced by the different honey samples on cell membrane potential, membrane integrity and metabolic activity were evaluated using *bis*-(1,3-dibutylbarbituric acid) trimethine oxonol (DiBAC4 (3); Sigma-Aldrich, Munich, Germany), propidium iodide (PI; Sigma-Aldrich, Munich, Germany) and calcein-AM (Sigma-Aldrich, Munich, Germany) respectively

All cytometric assays were performed using a standard FACS Calibur TM (BD Biosciences, Sidney, Australia) equipped with 3 photomultipliers (PMTs), standard filters, and a 15 mW, 488 nm argon laser, using Cell- Quest Pro software (version 4.0.2 BD Biosciences). Data was recorded for 20,000 cells for each assay.

### 4.5. Statistical Analysis

All experimental procedures were replicated three times. The results were expressed as mean values and the respective standard deviations. The statistical analysis was performed using IBM SPSS statistics v.24.0 (SPSS, Armonk, NY, USA) [41]. All variables were tested for the assumptions of normality and homoscedasticity. To evaluate if there were any differences between non-treated bacteria and honey treated bacteria at the different time points sampled in each assay, paired-sample Student’s t-test or Wilcoxon signed rank test were used. Moreover, to compare the differences between the two honey concentrations tested in each assay, unpaired-sample Student’s *t*-test or Mann–Whitney test were used, whereby *p* < 0.05 was considered to be significant. 

## 5. Conclusions

This research work contributes to reveal some of the mechanisms of antibacterial action of different types of honey, and to assess whether these mechanisms are similar or different between varieties and those described for MkH.

Our results demonstrate that the different honey samples induced quite similar effects on bacteria, consisting in a remarkable metabolic disruption as primary physiological effect upon *S. aureus*, as well as membrane potential imbalance and membrane injury upon both, *S. aureus* and *E. coli*. However, the depth of bacteria response throughout the treatment varied depending on the honey concentrations tested and among honey varieties, probably due to differences in their chemical composition.

The honey concentrations tested were not able to reduce, in all cases, cell viability, especially for *S. aureus*. Nevertheless, taking into account the relationship between the concentration of honey and the effect on bacteria, more studies are needed to verify whether higher concentrations than those tested are capable of producing bactericidal effects.

The results of this study could be the basis of future research works aiming to comprehend the antibacterial action mechanisms of different varieties of honey. 

## References

[1] Oniciuc E.A., Likotrafiti E., Alvarez-Molina A., Prieto M., López M., Alvarez-Ordóñez A. (2019). Food processing as a risk factor for antimicrobial resistance spread along the food chain. Curr. Opin. Food Sci..

[2] Surveillance of antimicrobial resistance in Europe (2017) (2018). Annual report of the European Antimicrobial Resistance Surveillance Network (EARS-Net).

[3] Bintsis T. (2017). Foodborne pathogens. AIMS Microbiol..

[4] Oryan A., Alemzadeh E., Moshiri A. (2016). Biological properties and therapeutic activities of honey in wound healing: A narrative review and meta-analysis. J. Tissue Viability.

[5] Deng J., Liu R., Lu Q., Hao P., Xu A., Zhang J., Tan J. (2018). Biochemical properties, antibacterial and cellular antioxidant activities of buckwheat honey in comparison to manuka honey. Food Chem..

[6] Brudzynski K., Miotto D., Kim L., Sjaarda C., Maldonado-Alvarez L., Fukś H. (2017). Active macromolecules of honey form colloidal particles essential for honey antibacterial activity and hydrogen peroxide production. Sci. Rep..

[7] Fyfe L., Okoro P., Paterson E., Coyle S., McDougall G.J. (2017). Compositional analysis of Scottish honeys with antimicrobial activity against antibiotic-resistant bacteria reveals novel antimicrobial components. LWT - Food Sci. Technol..

[8] Liu M.Y., Cokcetin N.N., Lu J., Turnbull L., Carter D.A., Whitchurch C.B., Harry E.J. (2018). Rifampicin-Manuka Honey Combinations Are Superior to Other Antibiotic-Manuka Honey Combinations in Eradicating Staphylococcus aureus Biofilms. Front. Microbiol..

[9] Liu M., Lu J., Müller P., Turnbull L., Burke C.M., Schlothauer R.C., Carter D.A., Whitchurch C.B., Harry E.J. (2015). Antibiotic-specific differences in the response of Staphylococcus aureus to treatment with antimicrobials combined with manuka honey. Front. Microbiol..

[10] Jenkins R., Cooper R. (2012). Improving Antibiotic Activity against Wound Pathogens with Manuka Honey In Vitro. PLoS ONE.

[11] Bucekova M., Jardekova L., Juricova V., Bugarova V., Di Marco G., Gismondi A., Leonardi D., Farkasovska J., Godocikova J., Laho M. (2019). Antibacterial Activity of Different Blossom Honeys: New Findings. Molecules.

[12] Poli J.P., Guinoiseau E., Luciani A., Yang Y., Battesti M.J., Paolini J., Costa J., Quilichini Y., Berti L., Lorenzi V. (2018). Key role of hydrogen peroxide in antimicrobial activity of spring, Honeydew maquis and chestnut grove Corsican honeys on Pseudomonas aeruginosa DNA. Lett. Appl. Microbiol..

[13] Roberts A.E.L., Brown H.L., Jenkins R. (2015). On the antibacterial effects of manuka honey: Mechanistic insights. Res. Rep. Biol..

[14] Roberts A.E.L., Maddocks S.E., Cooper R.A. (2015). Manuka honey reduces the motility of Pseudomonas aeruginosa by suppression of flagella-associated genes. J. Antimicrob. Chemother..

[15] Henriques A.F., Jenkins R.E., Burton N.F., Cooper R.A. (2011). The effect of manuka honey on the structure of Pseudomonas aeruginosa. Eur. J. Clin. Microbiol. Infect. Dis..

[16] Henriques A.F., Jenkins R.E., Burton N.F., Cooper R.A. (2010). The intracellular effects of manuka honey on Staphylococcus aureus. Eur. J. Clin. Microbiol. Infect. Dis..

[17] Combarros-Fuertes P., Estevinho L.M., Dias L.G., Castro J.M., Tomás-Barberán F.A., Tornadijo M.E., Fresno-Baro J.M. (2019). Bioactive Components and Antioxidant and Antibacterial Activities of Different Varieties of Honey: A Screening Prior to Clinical Application. J. Agric. Food Chem..

[18] Salonen A., Virjamo V., Tammela P., Fauch L., Julkunen-Tiitto R. (2017). Screening bioactivity and bioactive constituents of Nordic unifloral honeys. Food Chem..

[19] Liu J.R., Ye Y.L., Lin T.Y., Wang Y.W., Peng C.C. (2013). Effect of floral sources on the antioxidant, antimicrobial, and anti-inflammatory activities of honeys in Taiwan. Food Chem..

[20] Carter D.A., Blair S.E., Cokcetin N.N., Bouzo D., Brooks P., Schothauer R., Harry E.J. (2016). Therapeutic Manuka Honey: No Longer So Alternative. Front. Microbiol..

[21] Li L., Mendis N., Trigui H., Oliver J.D., Faucher S.P. (2014). The importance of the viable but non-culturable state in human bacterial pathogens. Front. Microbiol..

[22] Oliver J.D. (2010). Recent findings on the viable but nonculturable state in pathogenic bacteria. FEMS Microbiol. Rev..

[23] Ducret A., Chabalier M., Dukan S. (2014). Characterization and resuscitation of ‘non-culturable’ cells of Legionella pneumophila. BMC Microbiol..

[24] Zandri G., Pasquaroli S., Vignaroli C., Talevi S., Manso E., Donelli G., Biavasco F. (2012). Detection of viable but non-culturable staphylococci in biofilms from central venous catheters negative on standard microbiological assays. Clin. Microbiol. Infect..

[25] Pasquaroli S., Zandri G., Vignaroli C., Vuotto C., Donelli G., Biavasco F. (2013). Antibiotic pressure can induce the viable but non-culturable state in Staphylococcus aureus growing in biofilms. J. Antimicrob. Chemother..

[26] Packer J.M., Irish J., Herbert B.R., Hill C., Padula M., Blair S.E., Carter D.A., Harry E.J. (2012). Specific non-peroxide antibacterial effect of manuka honey on the Staphylococcus aureus proteome. Int. J. Antimicrob. Agents.

[27] Sträuber H., Müller S. (2010). Viability states of bacteria-Specific mechanisms of selected probes. Cytom. Part A.

[28] Combarros-Fuertes P., Estevinho L.M., Teixeira-Santos R., Rodrigues A.G., Pina-Vaz C., Fresno J.M., Tornadijo M.E. (2019). Evaluation of Physiological Effects Induced by Manuka Honey Upon Staphylococcus aureus and Escherichia coli. Microorganisms.

[29] Jenkins R., Burton N., Cooper R. (2011). Manuka honey inhibits cell division in methicillin-resistant Staphylococcus aureus. J. Antimicrob. Chemother..

[30] Tracy B.P., Gaida S.M., Papoutsakis E.T. (2010). Flow cytometry for bacteria: Enabling metabolic engineering, synthetic biology and the elucidation of complex phenotypes. Anal. Biotechnol..

[31] Sousa J.M., de Souza E.L., Marques G., Meireles B., de Magalhães Cordeiro Â.T., Gullón B., Pintado M.M., Magnani M. (2016). Polyphenolic profile and antioxidant and antibacterial activities of monofloral honeys produced by Meliponini in the Brazilian semiarid region. Food Res. Int..

[32] Tomás-Barberán F.A., Martos I., Ferreres F., Radovic B.S., Anklam E. (2001). HPLC flavonoid profiles as markers for the botanical origin of European unifloral honeys. J. Sci Food Agric..

[33] Ferreres F., Andrade P., Tomás-Barberán F.A. (1996). Natural Occurrence of Abscisic Acid in Heather Honey and Floral Nectar. J. Agric. Food Chem..

[34] Truchado P., Martos I., Bortolotti L., Sabatini A.G., Ferreres F., Tomas-Barberan F.A. (2009). Use of Quinoline Alkaloids as Markers of the Floral Origin of Chestnut Honey. J. Agric. Food Chem..

[35] García-Tenesaca M., Navarrete E., Iturralde G., Villacrés Granda I., Tejera E., Beltrán-Ayala P., Giampieri F., Battino M., Alvarez-Suarez J. (2018). Influence of Botanical Origin and Chemical Composition on the Protective Effect against Oxidative Damage and the Capacity to Reduce In Vitro Bacterial Biofilms of Monofloral Honeys from the Andean Region of Ecuador. Int. J. Mol. Sci..

[36] Combarros-Fuertes P., Valencia-Barrera R.M., Estevinho L.M., Dias L.G., Castro J.M., Tornadijo M.E., Fresno J.M. (2019). Spanish honeys with quality brand: A multivariate approach to physicochemical parameters, microbiological quality and floral origin. J. Apic. Res..

[37] Cushnie T.P.T., Lamb A.J. (2011). Recent advances in understanding the antibacterial properties of flavonoids. Int. J. Antimicrob. Agents.

[38] Ahmad A., Kaleem M., Ahmed Z., Shafiq H. (2015). Therapeutic potential of flavonoids and their mechanism of action against microbial and viral infections—A review. Food Res. Int..

[39] Cooper R.A., Jenkins L., Henriques A.F.M., Duggan R.S., Burton N.F. (2010). Absence of bacterial resistance to medical-grade manuka honey. Eur. J. Clin. Microbiol. Infect. Dis..

[40] Maddocks S.E., Jenkins R.E. (2013). Honey: A sweet solution to the growing problem of antimicrobial resistance?. Future Microbiol..

[41] IBM (2016). IBM SPSS Statistics for Windows, Version 24.0.

